# Transcutaneous electrical vagus nerve stimulation to suppress premature ventricular complexes (TREAT PVC): study protocol for a multi-center, double-blind, randomized controlled trial

**DOI:** 10.1186/s13063-023-07713-2

**Published:** 2023-10-23

**Authors:** Cheng Cai, Nan Wu, Gang Yang, Shu Yang, Wenjie Liu, Minglong Chen, Sunny S. Po, on behalf of the TREAT PVC investigators

**Affiliations:** 1 Division of Cardiology, The First Affiliated Hospital of Nanjing Medical University, Nanjing, 210029 China; 2 Heart Rhythm Institute, Section of Cardiovascular Diseases, The University of Oklahoma Health Sciences Center, Oklahoma, USA

**Keywords:** Premature ventricular complexes, Neuromodulation, Tragus stimulation, Randomized controlled trial

## Abstract

**Background:**

The autonomic nervous system can be responsible for the initiation and maintenance of arrhythmias. Low-level tragus stimulation (LLTS), a noninvasive form of autonomic neuromodulation, has been shown to be effective in treating atrial fibrillation. We intended to treat frequent premature ventricular complexes (PVCs) with LLTS.

**Methods and design:**

The present study will be a prospective multicenter, double-blind, randomized, controlled trial to assess the antiarrhythmic effects of LLTS on frequent PVCs in patients without structured heart disease (SHD). A total of 100 patients with PVC burden > 10% will be randomly assigned to the active or sham LLTS in 1:1 fashion and receive the proposed intervention for 6 months. The primary outcome is PVC burden at 6 months as assessed by 10 days of continuous ambulatory electrocardiographic monitoring. Secondary outcomes include heart rate variability (HRV), quality of life, skin sympathetic nerve activity, and inflammatory markers. Adverse events will also be recorded.

**Discussion:**

The present trial will be the first to evaluate the effect of LLTS on frequent PVCs on patients without SHD. LLTS may serve as a low-cost, minimal-risk, and non-invasive alternative to conventional antiarrhythmic therapy.

**Trial registration:**

ClinicalTrial.gov NCT04909528. Registered on 17 June 2021. World health organization trial registration data set was shown in Supplementary Table 1.

**Supplementary Information:**

The online version contains supplementary material available at 10.1186/s13063-023-07713-2.

## Background

Premature ventricular complexes (PVCs) are the most common arrhythmias in clinical practice with an estimated prevalence of 1 to 4% in the general population on standard 12-lead electrocardiography and 40–75% of healthy individuals assessed by 24-h and 48-h ambulatory monitoring [1, 2]. The general impression is that PVCs in patients without structural heart disease (SHD) are benign [2, 3]. However, studies consistently demonstrated that PVCs could trigger malignant ventricular arrhythmias or even sudden cardiac death, especially in patients suffered from myocardial infarction (MI) [4, 5]. Additionally, frequent PVCs may be the primary etiology for PVC-induced cardiomyopathy [6] and lead to worsening of left ventricular dysfunction in patients with pre-existing cardiomyopathy [7].

Pharmacological therapies, including β-blockers and calcium channel blockers, are often prescribed as first-line treatment for patients with symptomatic or frequent PVCs [8, 9]. However, β-blockers or calcium channel blockers alone or their combination offer relief in less than 25% patients [10, 11]. Medical therapy may be discontinued due to lack of efficacy or intolerable side effects. Catheter ablation has also been recommended as first-line therapy for PVCs but can sometimes be challenging because of the anatomical obstacles [8, 9]. The other concern with this invasive approach is the rare but severe complications including aortic dissection, atrioventricular block, myocardial infarction, cardiac tamponade, and stroke [12].

Recent evidence suggests that the autonomic system can be responsible for arrhythmogenesis and arrhythmia maintenance. Invasive approaches, such as stellate ganglion block and cardiac sympathetic denervation, have shown antiarrhythmic effects in patients with refractory ventricular arrhythmias (VA) [13–15]. Continuous vagal stimulation reduced VA and prevented sudden cardiac death in a canine model of post-myocardial infarction [16]. It is widely accepted that increased cardiac sympathetic activity plays an important role in the generation of VAs; suppression of cardiac sympathetic tone and/or stimulation of cardiac parasympathetic tone may have antiarrhythmic effects on VAs. Low-level tragus stimulation (LLTS) targets the auricular branch of the vagus nerve, an afferent nerve that relays information to central vagal projections in the brain stem. The signal is processed in the brain stem and higher centers, which in turn provide the efferent neural signal to the heart, which reaches the target organ via the vagus nerve. In addition to the scientific basis for the antiarrhythmic effect, previous clinical trials have supported that LLTS is a non-invasive and safe alternative for the treatment of atrial fibrillation [17, 18]. There have been at least two animal models have demonstrated the antiarrhythmic effect of LLTS on VAs induced by MI [19, 20]. Currently, the chronic effects of LLTS on VAs in humans remain unknown. Therefore, we designed a multicenter, double-blind, randomized, controlled trial between an LLTS group and a sham-control group, both receiving standard antiarrhythmic drug treatment, to: (1) assess the antiarrhythmic effects of LLTS on frequent PVCs in patients without SHD, (2) explore the possible mechanism of LLTS responsible for its antiarrhythmic effects, and (3) identify the potential responders to this novel therapeutic strategy.

## Methods/design

### Study design

**Fig. 1:**
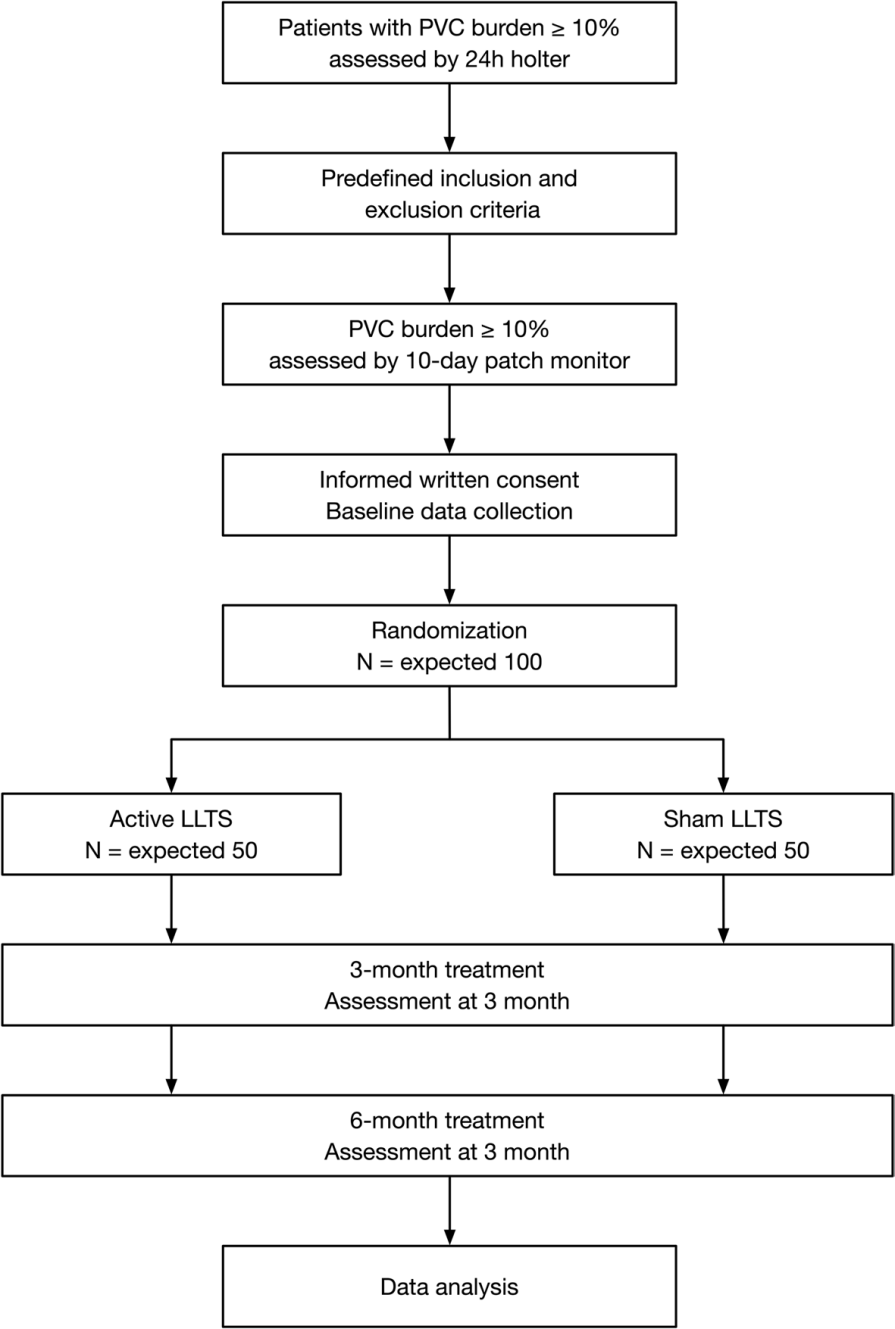
Flowchart of the prospective trial. LLTS indicates low-level tragus stimulation, PVC premature ventricular complexes

**Fig. 2:**
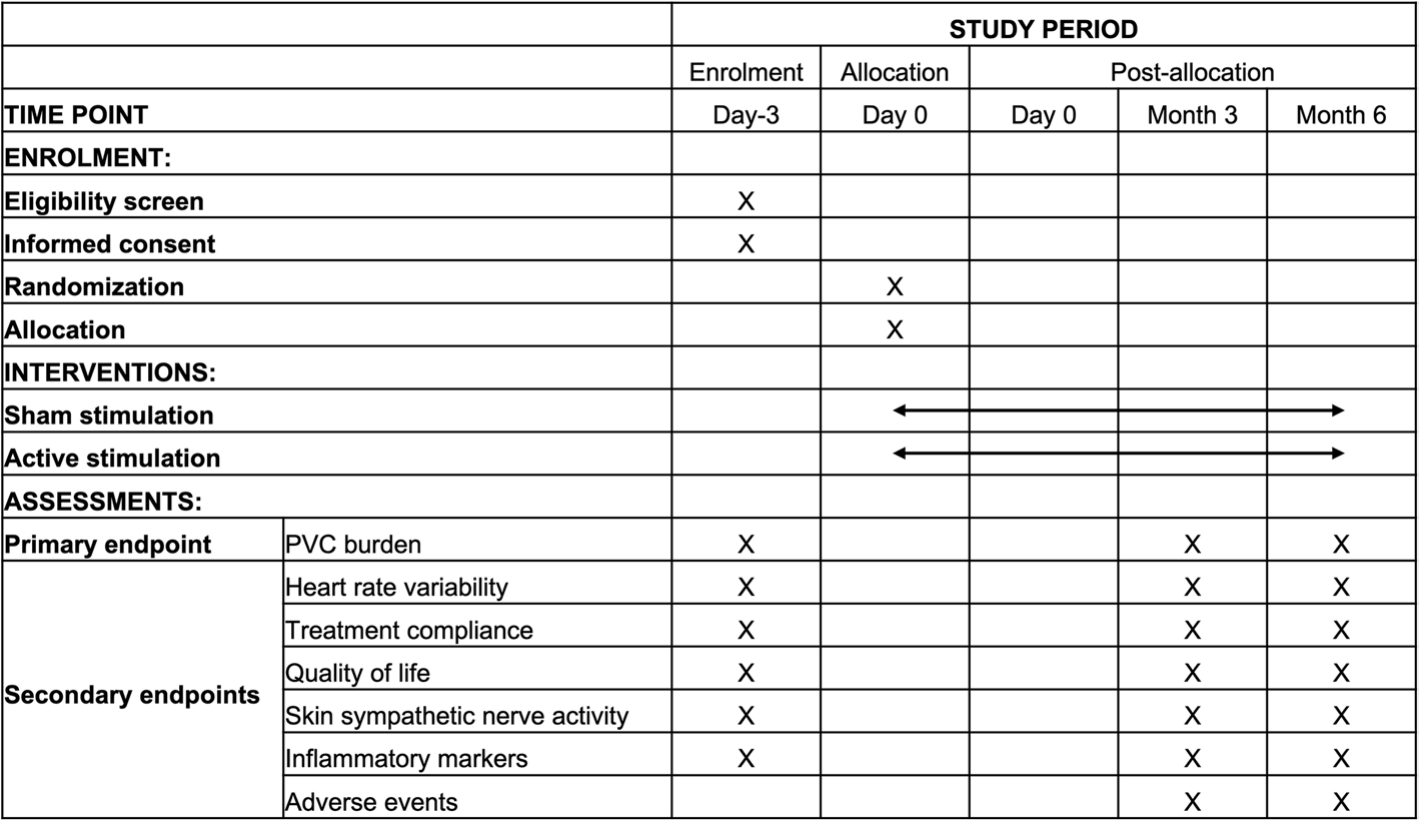
Time schedule of enrolment, interventions, assessments and visits for participants. EKG indicates electrocardiogram, LLTS low-level tragus stimulation, SKNA skin sympathetic nerve activity

The transcutaneous electrical vagus nerve stimulation to suppress premature ventricular complexes (TREAT PVC) trial is a prospective, multicenter, double-blind, sham-controlled, randomized clinical trial (ClinicalTrials.gov Identifier: NCT04909528). The design of the TREAT PVC trial is summarized in Fig. 1. Briefly, all eligible subjects who have given informed consent will be randomly assigned to active or sham LLTS in a 1:1 fashion and will receive the proposed intervention for 6 months. Primary and secondary outcomes will be measured at 3 and 6 months. The timetable for enrolment, intervention, and assessments is shown in Fig. 2.

### Participants and eligibility criteria

#### Inclusion criteria

- Age of 18 to 80 years
- Symptomatic PVCs refractory to ≥ 1 antiarrhythmic drugs (including β-blockers and calcium-channel blockers)
- PVC burden ≥ 10%
- Arrhythmias originated from any focus (foci) in the right ventricular or left ventricular

#### Exclusion criteria

- Ejection fraction (EF) < 45% unless proven to be PVC-mediated cardiomyopathy (history of improving LVEF by > 15% when PVC burden was reduced by pharmacological agents or ablation)
- EF continues to decrease in the past 4 months regardless of the etiology
- Unwilling to continue current pharmacological therapy during the study period (6 months)
- Severe heart failure with NYHA Class ≥ III
- VAs attributed to underlying structural heart disease, known myocardial scar or myocarditis
- Change the dosing of antiarrhythmic drug, including β-blockers and calcium channel blockers, within 2 months prior to enrollment
- Unsuccessful ablation within 3 months before recruitment
- Patients are on amiodarone
- Patients with known thyroid issues or renal dialysis
- Life expectancy of < 12 months.

### Recruitment and enrollment

Recruitment will be conducted through the electrophysiology clinic of the First Affiliated Hospital of Nanjing Medical University and all other participating hospitals. Consecutive patients with frequent PVC will be screened for eligibility and approached for participation in the study. In addition, an investigator-handbook and training events will be conducted for referring physicians in the study to achieve adequate participant enrolment. Specifically, we will identify those patients who have a PVC burden of at least 10% measured by 24-h holter after correcting for some common reversible causes for PVC, such as caffeine intake, electrolyte imbalance, and staying up late. For patients who agree to consider participation, a member of the study team will describe the potential risks/benefits, outline the study interventions, and conduct recruitment assessments. For willing and eligible patients, we offer a 10-day single-lead electrocardiogram (ECG) patch monitor to accurately determine their PVC burden.

### Randomization and blinding

After baseline measurements have been collected, patients will be randomized 1:1 to active or sham LLTS using a central computerized randomization system to ensure blocked randomization. Sequence generation will be performed by an independent researcher through R, with a 1:1 allocation. To guarantee allocation concealment and to avoid selection bias, an independent researcher not involved in the trial will be charged to the custody of the sequences generated and to the allocation of the participants to each condition. Patients will not be informed of which stimulation site (tragus vs. earlobe) provides active stimulation. The investigators who assess the outcomes are also blinded to treatment allocation. The research coordinators who instruct the participant on ear clip placement according to randomized assignment and the study statistician are unblinded.

Unblinding can be performed at any time if participants experience an emergency. If necessary to ensure adequate treatment, where the principal investigator deems it necessary to identify the treatment, the investigator may perform unblinding and record the breach of blinding.

### Study intervention

#### Intervention description

**Fig. 3:**
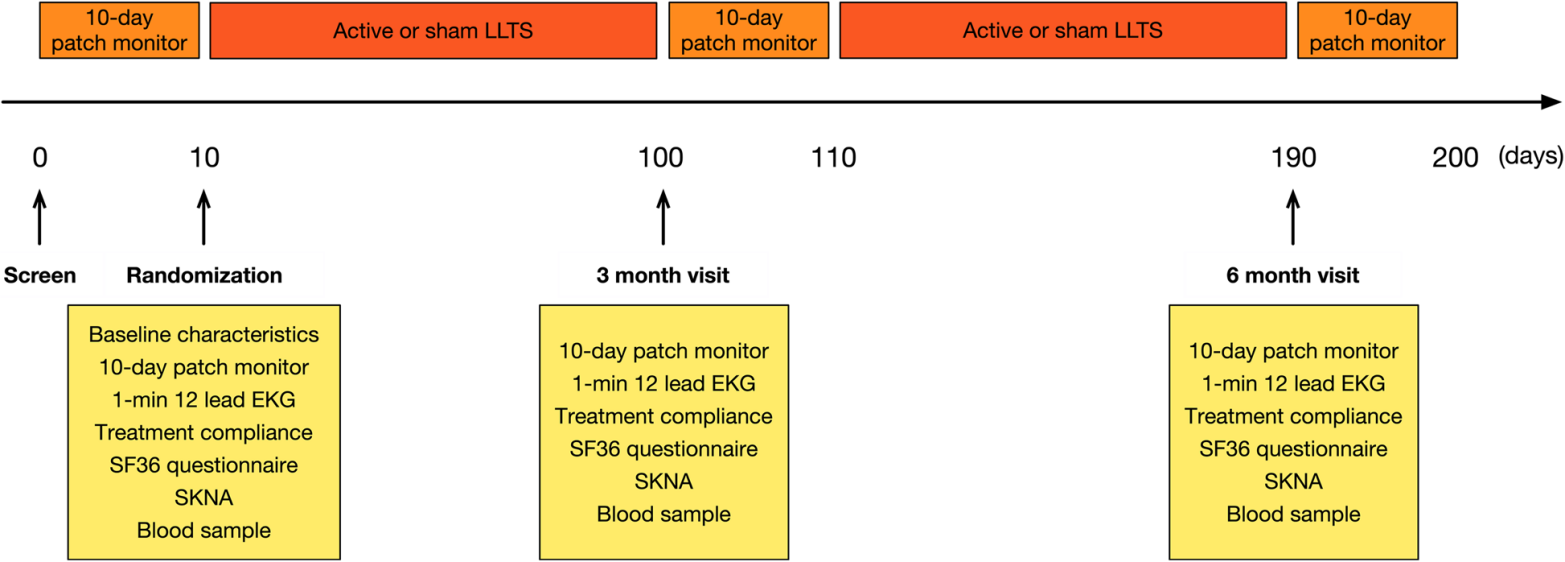
Real photo of how LLTS is applied

For the patients allocated to the active stimulation groups, LLTS is delivered through a transcutaneous electrical nerve stimulation device (Parasym device, Parasym Health, Inc, London, UK) by attaching an ear clip to the tragus, which is innervated by the auricular branch of the vagus nerve (Fig. 3). In the sham group, stimulation was delivered to the earlobe, which is devoid of vagal innervation. Subjects in both groups are instructed to stimulate the tragus or earlobe twice a day for half an hour each time. The left and right tragi/earlobes will be stimulated alternatively. The device is set to a pulse width of 200 μs and a pulse frequency of 20 Hz, while the stimulation intensity is individually titrated to an amplitude above the threshold of perception and 1 mA below the level of discomfort.

#### Criteria for discontinuing or modifying allocated interventions

As a non-invasive treatment, LLTS carries a very low risk of potential harm. Adverse events may include, but are not limited to, hypotension, bradycardia, fainting, severe pain, and local infection. All adverse events and their management will be recorded from baseline to 6 months. Circumstances in which individual participants may discontinue or modify the intervention include the occurrence of intolerable or unexpected side effects during the course of the therapy, and other circumstances that would endanger participant’s health if they continued in the study, as well as at participant request.

#### Relevant concomitant care and interventions permitted or prohibited during the trial

During the trial period, existing drug therapy will be continued with minimal changes possible. A change of medication during the treatment is not prohibited, but will be recorded as an event that requires additional modification.

### Follow-up schedule

**Fig. 4:**
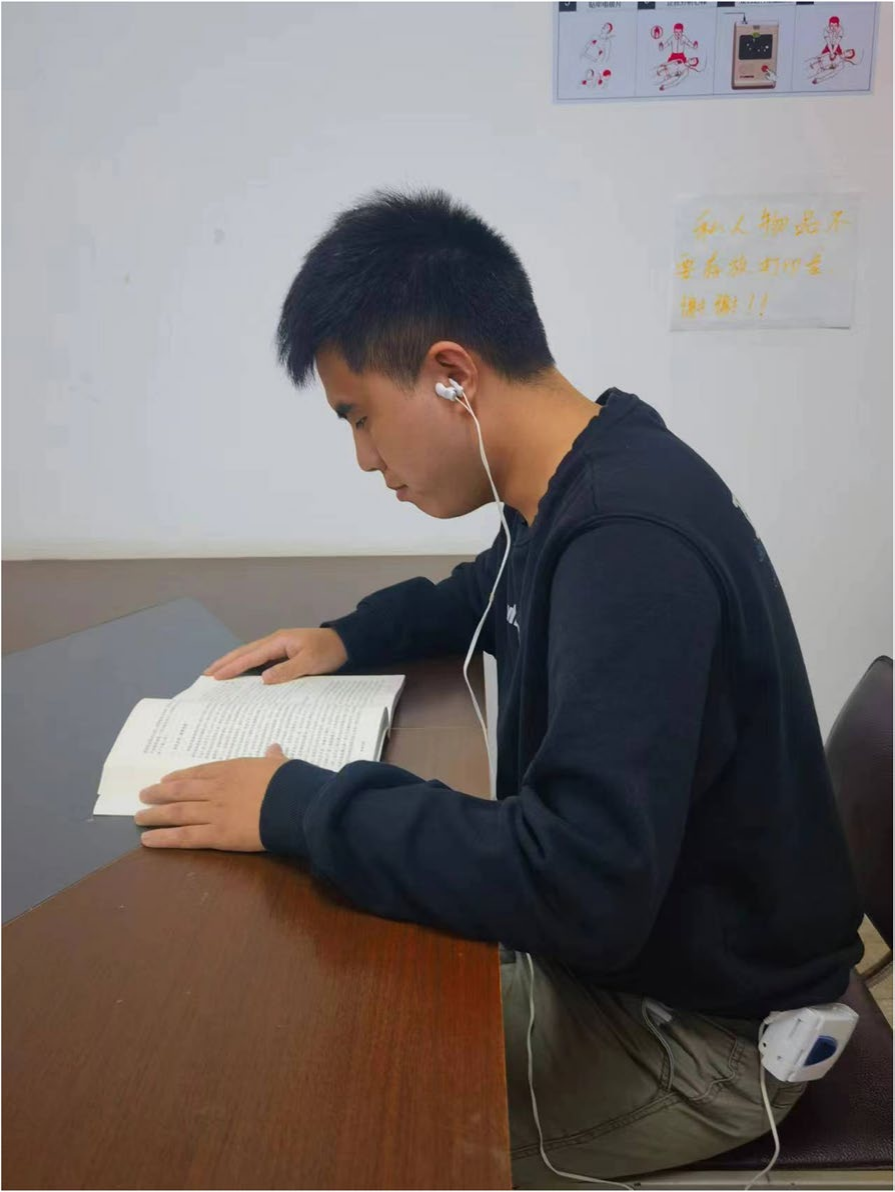
Schedule of enrollment, intervention, and assessments (SPIRIT figure). Patients with frequent PVC were randomized into sham (no chronic LLTS) and active (chronic LLTS) groups. Ten days of continuous ECG monitoring at baseline and 3 and 6 months was performed to calculate PVC burden before and after LLTS treatment. In addition, 1 min of ECG, treatment compliance, 36SF questionnaire, SKNA, and blood samples were collected at baseline and 3- and 6-month follow-up visits. ECG indicates electrocardiogram, LLTS low-level tragus stimulation, PVC premature ventricular complexes, SKNA skin sympathetic nerve activity

The planned follow-up period is 6 months, and two outpatient visits are scheduled at 3 and 6 months over this treatment duration. The assessments performed at each visit are displayed in Fig. 4.

### Primary outcome measures

#### PVC burden

At baseline and 3 and 6 months, patients will receive 10 days of continuous ECG monitoring to evaluate their PVC burden (reported as the percentage of all recorded beats that are PVCs throughout the study) using an adhesive ECG patch monitor (CardioGuard, Zhengxin Information Technology Co. Ltd., Shanxi, China). The P waves and QRS complexes were automatically classified and manually verified as normal sinus rhythm, premature atrial complexes or PVCs, or noise by comparison with adjacent waves. Each QRS complex in episodes of non-sustained ventricular tachycardia will be counted as a PVC. The mean PVC burden at each time point will be used to test the antiarrhythmic effect of LLTS on PVCs.

### Secondary outcomes measures

#### Heart rate variability (HRV)

HRV will be generated while monitoring PVC burden with the monitor patch to quantitatively evaluate the balance between the sympathetic and vagal nerves at baseline and 3 and 6 months. The R-R intervals will be derived from the adjacent normal sinus beats (i.e., N–N intervals). Time domain measurements, including SDNN, SDNN index, root mean square of successive R-R interval differences (RMSSD), the proportion of NN50 (the number of pairs of successive R-R intervals that differ by more than 50 ms) divided by the total number of R-R intervals (pNN50) will be calculated automatically. The power spectrum including very low frequency (LF) power (0.01–0.05 Hz), LF power (0.05–0.15 Hz), and high frequency (HF) power (0.15–0.5 Hz) will be estimated by Welch’s averaged periodogram method. The LF/HF ratio will also be calculated and considered an index of sympathetic–parasympathetic balance. The means of these HRV parameters will be used to determine the effect of LLTS on autonomic nervous system.

#### Treatment compliance

Patients will be requested to keep a daily log of the time and duration of LLTS application, amplitude settings, and any discomfort associated with each daily session. The cumulative number of stimulation hours recorded by the stimulator will be correlated with the daily log as an objective measure of compliance to the stimulation protocol.

#### Quality of life

Self-rated mental health will be measured using the SF-36 questionnaire, mental component score, at baseline and after 3 and 6 months. The scale was translated into Chinese using a two-part method that included (a) translation by bilingual investigators and (b) back translation. The two versions (English and Chinese) of the instrument were compared and verified by a qualified, bilingual expert specializing in the design and cross-cultural validation of study questionnaires. The specific numerical values for each parameter will be compared.

#### Skin sympathetic nerve activity (SKNA)

This refers to the electrical activity originating from the nerve structure that can be measured on the surface of the skin to reflect sympathetic tone [21] and is recorded using three standard ECG patch electrodes and a prototype device with a wide bandwidth (16kHz) and high sample rate (10kHz). The three electrodes include two recording electrodes and one reference lead to form a bipolar recording channel. One pair of electrodes are placed in the right and left subclavicular areas as the negative and positive leads, respectively, and the third lead is placed below the right arcus costalis as the reference. The recorded electrical signals are analyzed by using custom written software, amplified and band-pass filtered (500–1000 Hz) to display SKNA. We also create an algorithm to quantify SKNA by calculating the eSKNA, which can indicate periods of nerve activation in the given time period. SKNA will be continuously recorded for 30 min at baseline and 3 and 6 months follow-up.

#### Inflammatory markers

Blood samples (10 ml) will be collected at baseline and at 3 and 6 months for measurement of inflammatory cytokines. The blood was then centrifuged to separate the serum and stored at − 80℃ in a refrigerator. Serum levels of inflammatory cytokines, including interleukin-1β, interleukin-6, and TNF-α, were measured using commercially available multiplex assays. All immunoassays will run in duplicate and read according to the manufacturer’s instructions. The investigators performing the cytokine assays will be blinded to group allocation.

### Adverse events

Any adverse events and their management will be recorded from baseline to 6 months. These adverse events may include, but are not limited to, hypotension, bradycardia, fainting, severe pain, and local infection.

### Data collection and management

#### Plans for assessment and collection of outcomes

All investigators will be trained in terms of the study protocol, the SF-36 questionnaire, and SKNA assessment execution. The data collection form (both paper-based and electronic case report form) will be provided for the entire research team. The first-time data collection by investigators will be conducted under the supervision of the principal investigators. Serum will be stored in a − 80℃ freezer with documented in a freezer log until used.

#### Plans to promote participant retention and complete follow-up

The investigators will make every effort to improve participants’ adherence to follow-up and treatment by providing routine health care online or by telephone. All study patients, except those who withdraw consent, will be included in the analysis during the course of treatment.

#### Data management

Each trial participant is assigned a unique eight-digit identification number that cannot be traced back to their proprietary information. Participant data, coded using these identification numbers, are stored in a central database which is a secure web-based platform designed for clinical trials. Clinical data are collected and input by trial staff. The principal investigator is responsible for ensuring the accuracy and completeness. During the study period, the principal investigator and a trained sub-investigator will have full access to the trial data. In addition, the monitor including the steering committee and data safety monitoring board will have access to the full dataset.

#### Confidentiality

All the participant data will be regarded as confidential. The investigator and members of research team must not disclose any information without prior written permission from the sponsor.

### Sample size calculation

Based on the 11 patients who underwent 1 month of stimulation, the average burden at baseline was 21.7% and the relative reduction after stimulation was 16.4%. We assume a baseline burden of 15% and expect a reduction of 40% at the end of 6 months. A sample size of 90 patients would provide at least 80% power to detect the specified effect sizes at a two-sided significance alpha level of 0.05. The sample size was calculated by using online statistic system (STATBOX, http://www.cnstat.org/statbox/; last access 9/19/2023). The exact formula used for sample size was as follows.

$$1-{\varvec{\beta}}={\varvec{P}}{\varvec{\upgamma}}[{\varvec{F}}(1,{\varvec{N}}-2,{\varvec{N}}\{\left({{\varvec{\upomega}}}_{1}{{\varvec{\upomega}}}_{2}\right)[\frac{{{\varvec{\mu}}}_{1}-{{\varvec{\mu}}}_{2}}{{\varvec{\sigma}}}{]}^{2}\})\ge {{\varvec{F}}}_{\boldsymbol{\alpha }}]$$

$${{\varvec{n}}}_{1}={{\varvec{N}}}^{\boldsymbol{*}}{{\varvec{\omega}}}_{1},\boldsymbol{ }{{\varvec{n}}}_{2}=\boldsymbol{ }{{\varvec{N}}}^{\boldsymbol{*}}{{\varvec{\omega}}}_{2}$$

To account for a potential drop-out rate of 10%, 50 patients in each group and 100 patients in total are planned to be recruited.

### Statistical plan

Descriptive statistics will be presented for baseline characteristics. Continuous variables are presented as mean ± SD or median (interquartile range), as appropriate. Categorical variables will be presented as proportions. Baseline variables will be compared between groups using Student’s *t*-test or Mann–Whitney *U* test for continuous variables and chi-square test for categorical variables. We will use a generalized estimating equation modelling approach to compare the outcome measure at the 3- and 6-month visits between the two groups after adjusting for the baseline measure [18]. Estimates reflect the exponentiated regression model terms and are interpreted as the ratio of the median responses in the active arm relative to the median response in the sham arm. Patients who discontinue the stimulation protocol and receive PVC ablation during the study period will be censored. These patients will be regarded as withdrawals from the study and the data from the last clinical follow-up will be included into final analysis.

Linear regression will be used to compare the association between the change in PVC burden at follow-up and measures of HRV and SKNA. Statistical significance will be set at *P* < 0.05 (two-sided). All statistical analyses will be performed using SAS software version 9.3 (SAS Institute, Cary, North Carolina).

### Interim analyses

There is no plan for interim analyses.

### Methods for additional analyses

Subgroup analyses may be performed to determine which group of PVC patients is likely to respond to LLTS. Additional subgroup analyses, based on emerging research questions of interest, may be conducted as well.

### Methods in analysis to handle protocol non-adherence and any statistical methods to handle missing data

The analyses will be done in intention-to-treat population including all patients who received intervention or sham intervention without major protocol deviations. Significant protocol deviations or complications (hypotension, bradycardia, fainting, severe pain, and local infection) and subsequent failure to obtain primary and secondary outcome data will be documented prior the database closure. In case of withdrawals and missing data, the last observation carried forward procedure will be applied where appropriate.

### Ethics and dissemination

The study protocol was approved by the Institutional Review Board of the First Affiliated Hospital of Nanjing Medical University, as well as by the ethics committee at each center. The study will be conducted according to the principles of the Declaration of Helsinki, and all subjects provide written informed consent before the enrollment. The results of this trial will be submitted for publication in a peer-reviewed journal after study completion and finalization of the study report. There are no publication restrictions. The principal investigator, sub-investigator, the steering committee, and investigators in all participating hospitals will be listed as authors of the trial result reports. No professional writers will be hired. The informed consent documents will be available from the corresponding author upon reasonable request.

### Protocol amendments

Amendments for any change to the existing protocol that significantly affects the scopes of the investigation, or the scientific quality of the study will be submitted to the Institutional Review Board before the amendment release.

### Ancillary and post-trial care

As there is no expected harm from participation in the trial, no specific provision for post-trial or ancillary care is foreseen by the study protocol.

### Oversight and monitoring

#### Steering committee

The steering committee will meet prior to the study initiation and every 6 months thereafter. The principal responsibilities include overall supervision of the trial, taking steps to reduce deviations from the protocol and reviewing the safety data.

#### Data safety monitoring board (DSMB)

The role of data safety monitoring board was to safeguard the interests of the trial’s participants and to monitor the data collected in the trials. All the adverse effects will be reported to DSMB formed by three cardiologists not involved in the trial. The DSMB has the authority to terminate the trial if there are concerns about safety or efficacy.

## Discussion

### Significance

TREAT PVC trial will contribute to the current literature at multiple levels. Clinically, it will be the first study to investigate whether LLTS can reduce the PVC burden in patients without SHD who suffer from frequent PVCs. LLTS may turn out to be a low-cost, minimal-risk, non-invasive alternative to antiarrhythmic or ablative treatment for these patients. Scientifically, the trial may shed new light on the mechanisms underlying the antiarrhythmic effects of LLTS. Previous literature supported the hypothesis that the antiarrhythmic effect of LLTS was attributed to its anti-adrenergic and possibly anti-inflammatory effects [17, 18]. To corroborate these hypotheses, we will collect data such as skin sympathetic nerve activity, heart rate variability, and inflammatory markers, such as interleukin-1β, interleukin-6, and tumor necrosis factor-α at baseline and during the treatment course, which may help elucidate the underlying antiarrhythmic mechanism. Additionally, these measurements will also help to clarify the correlation between autonomic perturbance and ventricular arrhythmias. Last but not least, these data will also help us to identify the responders to this neural therapy.

From the perspective of patients, the trial focuses not only on PVC burden but also on patients’ adherence and quality of life. As patient compliance is of paramount importance for the efficacy of the self-administrated treatment, it will be highly valuable to include the patients’ perspective in the development and evaluation of the intervention. A diary log and the SF-36 questionnaire will be introduced to assess the degree to which patients feel symptomatically improved by the intervention.

#### Limitation of the study design

The PVC burden was determined by 10-day patch monitoring, which might not accurately reflect total PVC burden. Currently, 7–14 days of monitoring is considered as reasonable compromise between acceptable levels of error and reduced variability in predicting total PVC burden.

Previous studies have shown that earlobe stimulation, in contrast to tragus stimulation, does not result in activation of central vagal projections, and is therefore a reasonable sham control [22]. However, it is possible that a patient may seek out and become aware of the site of active stimulation, affecting the double-blind nature of the study. We will search the internet using key words related to LLTS (in both Chinese and English) every 2 months to see how easy it is for a study subject to figure out if s/he is receiving sham or active stimulation. At the end of the study, each patient will be queried if s/he has unblinded himself or herself.

According to the latest guidelines, catheter ablation has been considered the first-line therapy for the patients with frequent and symptomatic PVCs in an otherwise normal heart, especially those whose PVCs originate from the right ventricular outflow tract. We acknowledge that those with polymorphic, likely epicardial or left-sided originated PVCs may be shifted to this neural therapy and cause bias.

In the present study, we will not stratify patients according to the participating centers. However, we will make some efforts to minimize the center effects in the conduction of the study, such as collecting data from different centers by electronic data capture system, developing standard operating procedures, analyzing ECG data in the core lab, and so on.

Finally, since the prospective trial will only last for 6 months and no further follow-up will be performed, the long-term effect of LLTS will not be investigated in the present study. However, we will encourage the subjects to have holter or other ECG monitoring after the cessation of the LLTS therapy to see if the effect is long-lasting.

## Trial status

This is an ongoing trial. Recruitment began on June 1, 2021. Enrollment is expected to be completed on October 1, 2022. The current protocol is version 6.0, March 15, 2022.

### Supplementary Information

**Additional file 1.** Supplementary Table 1.

## Data Availability

All data requests should be submitted to the corresponding authors (Drs. Minglong Chen and Sunny S. Po) for consideration. After publication, access to anonymized data might be granted for non-commercial research at the discretion of the corresponding author.

## Abbreviations

ECG: Electrocardiogram
EF: Ejection fraction
HF: High frequency
HRV: Heart rate variability
LF: Low frequency
LLTS: Low-level tragus stimulation
MI: Myocardial infarction
NN50: The number of pairs of successive R-R intervals that differ by more than 50 ms
pNN50: The proportion of NN50 divided by the total number of R-R intervals
PVC: Premature ventricular complexes
RMSSD: Root mean square of successive R-R interval differences
SDNN: Standard deviation of normal R-R interval
SHD: Structural heart disease
SKNA: Skin sympathetic nerve activity
TREAT PVC: Transcutaneous electrical vagus nerve stimulation to suppress premature ventricular complexes
VA: Ventricular arrhythmia
