# Supplementary material for: Transcutaneous electrical vagus nerve stimulation to suppress premature ventricular complexes (TREAT PVC): study protocol for a multi-center, double-blind, randomized controlled trial
Source: Trials. 2023 Oct 23;24:683. doi: 10.1186/s13063-023-07713-2 (PMC10591365; doi:10.1186/s13063-023-07713-2)
Supplement: Supplementary file 1 — Additional file 1. Supplementary Table 1. [file 13063_2023_7713_MOESM1_ESM.docx]

***Supplementary table 1***

***World Health Organization Trial Registration Data Set***

| ***Data category*** | ***Information*** |
| --- | --- |
| ***Primary registry and trial identifying number*** | ***ClinicalTrials.gov NCT04909528*** |
| ***Date of registration in primary registry*** | ***05/19/2021*** |
| ***Secondary identifying numbers*** | ***None*** |
| ***Source(s) of monetary or material support*** | ***None*** |
| ***Primary sponsor*** | ***Division of Cardiology, The First Affiliated Hospital of Nanjing Medical University, Nanjing, 210029, China*** |
| ***Secondary sponsor(s)*** | ***The Affiliated Hospital of Xuzhou Medical University***  ***Wuxi No. 2 People's Hospital***  ***The First People's Hospital of Changzhou***  ***Second Affiliated Hospital of Nantong University***  ***Affiliated Hospital of Nantong University***  ***Jiangsu Province Official Hospital*** |
| ***Contact for public queries*** | ***Cheng Cai, MD***  ***caichen8971@gmail.com*** |
| ***Contact for scientific queries*** | ***Cheng Cai, MD***  ***Division of Cardiology, The First Affiliated Hospital of Nanjing Medical University, Nanjing, 210029, China*** |
| ***Public title*** | ***Transcutaneous Electrical Vagus Nerve Stimulation to Suppress Premature Ventricular Complexes (TREAT PVC)*** |
| ***Scientific title*** | ***Transcutaneous Electrical Vagus Nerve Stimulation to Suppress Premature Ventricular Complexes (TREAT PVC): a multi-center, double-blind, randomized controlled trial*** |
| ***Countries of recruitment*** | ***China*** |
| ***Health condition(s) or problem(s) studied*** | ***Premature ventricular complexes, Neuromodulation, Tragus stimulation*** |
| ***Intervention(s)*** | ***Experimental Group: Low-level tragus stimulation***  ***Control Group: Sham stimulation*** |
| ***Key inclusion and exclusion criteria*** | ***Inclusion Criteria: 1. age of 18 to 80 years; 2. symptomatic PVCs refractory to ≥1 antiarrhythmic drugs (including β-blockers and calcium-channel blockers); 3. PVC burden ≥ 10%; 4. arrhythmias originated from any focus (foci) in the RV or LV.***  ***Exclusion Criteria: 1. EF < 45% unless proven to be PVC-mediated cardiomyopathy (history of improving LV ejection fraction by >15% when PVC burden was reduced by pharmacological agents or ablation); 2. EF continues to decrease in the past 4 months regardless of the etiology; 3. unwilling to continue current pharmacological therapy during the study period (6 months); 4. severe heart failure with NYHA Class ≥ III; 5. ventricular arrhythmias attributed to underlying structural heart disease, known myocardial scar or myocarditis; 6. change the dosing of anti-arrhythmic drug, including β-blockers and calcium channel blockers, within 2 months prior to enrollment; 7. unsuccessful ablation within 3 months before recruitment; 8. patients are on amiodarone; 9. patients with known thyroid issues, or renal dialysis; 10. life expectancy of < 12 months.*** |
| ***Study type*** | ***Multicenter, prospective, randomized control trial with sham-control*** |
| ***Date of first enrolment*** | ***7/10/2021*** |
| ***Target sample size*** | ***100*** |
| ***Recruitment status*** | ***Recruiting*** |
| ***Primary outcome(s)*** | ***PVC burden at 6 months*** |
| ***Key secondary outcomes*** | ***Heart rate variability (HRV), quality of life, skin sympathetic nerve activity and inflammatory markers*** |
